# The Impact of COVID-19 on Drug Use and Harm Reduction Programming in the Middle East and North Africa (MENA) Region: a Regional Consultation of Stakeholders and People Who Use Drugs

**DOI:** 10.1007/s11469-021-00500-7

**Published:** 2021-07-13

**Authors:** Marie Claire Van Hout, Patricia Haddad, Elie Aaraj

**Affiliations:** 1 Public Health Institute, Liverpool John Moore’s University, Liverpool, L32ET UK; 2 Middle East and North African Harm Reduction Association (MENAHRA), Beirut, Lebanon

**Keywords:** Middle East and North Africa, People who use drugs (PWUD), Harm reduction, COVID-19

## Abstract

The Middle East and North Africa (MENA) region has witnessed a slow but steady increase in the harm reduction response since 2016. It is likely that such gains are threatened by the impact of COVID-19. Very little is known about the health response and situation of people who use drugs (PWUD) during the pandemic in the region. A mixed method study was conducted by the MENA Harm Reduction Association (MENAHRA) to assess the situation of PWUD and impacts on harm reduction services during COVID-19. Twelve countries and two regional viewpoints responded to the survey. A virtual focus group was held with the MENA Network of People who Use Drugs (MENANPUD) focal points (*n* = 11). The study highlights how COVID-19 aggravated existing marginalization and stigmatization of PWUD and other key populations in the MENA region, with government level resourcing severely impacted by COVID-19. It further illustrates the commitment by harm reduction non-governmental organizations (NGOs) in diversifying their response, particularly through mobile outreach to drug hot spots, and the reliance of technology to support awareness raising, telemedicine, and medicine supplies. Positive shifts are observed in harm reduction policy by governments in some MENA countries and the continued commitment to support PWUD communities by existing harm reduction NGOs. Continued advocacy for and implementation of harm reduction responses at the domestic and regional levels should be underpinned by inclusion in state health emergency planning and disease control efforts, awareness raising around innovation and telemedicine to support health and NGO support systems and medicine supply chains, resourcing of NGOs, and provision of economic support for PWUD, disease surveillance, and research.

## Background

COVID-19 (SARS-CoV-2) was declared a pandemic on March 11, 2020 (WHO, 2020), with initial reports of the disease occurring in the Middle East and North African (MENA) region originating from Iran in late February (Karamouzian & Madani, 2020; Sharifi et al., 2020). At the time of writing (January 27, 2021), the World Health Organization Eastern Mediterranean Regional Office (WHO-EMRO) reports 5,566,708 confirmed cases to date with 131,886 deaths (World Health Organization Eastern Mediterranean Regional Office (WHO EMRO), 2021). Figures may be underestimated due to low testing capacity and case reporting across the region (Karamouzian & Madani, 2020). The situation is exacerbated by the divisive nature of the MENA region in terms of population density, resources, healthcare coverage, and regional instability, with many living in impoverished conditions, particularly centred on limited access to clean water, sufficient nutrition and sanitation, healthcare access, and education (Ibrahim et al., 2020; UNESCWA, 2020; World Bank, 2018). The region continues to be affected by continued instability, terror threats, conflict, migration, and displacement of people internally and externally (Karamouzian & Madani, 2020). Country level lockdown, public health, and human rights-based responses have further been unevenly distributed (Aljazeera, 2020; Karamouzian & Madani, 2020; Solomon, 2020). Health infrastructure in some MENA countries is severely impacted by conflict (Emmons, 2020; UNOCHA, 2019). Humanitarian responses are hampered by lockdowns, curfews, and logistical and financial challenges (Karamouzian & Madani, 2020).

The ongoing humanitarian emergencies in the MENA region, conflict and economic migration, and internal/external displacement of large populations and the geographies of high-risk behaviours and key populations in the MENA region present massive challenges for public health systems in general and HIV and drug use prevention, treatment, and care programmes in particular. The complexities of disease transmission in the MENA region are not restricted to COVID-19 and include the blood borne viruses, human immune deficiency virus (HIV), and hepatitis C virus (Chemaitelly et al., 2019; Mumtaz et al., 2018; Van Hout & Aaraj, 2020). MENA countries are diverse in terms of geography, population size and demographics, characteristics of key populations, nature and extent of the blood borne virus problem, and scale of harm reduction implementation (MENAHRA, 2020a). There is considerable HIV incidence among people who inject drugs (PWID) in the MENA region, with more than one-third of HIV infections reported in 2019 attributed to PWID. Moreover, key populations such as men who have sex with men (MSM), commercial sex workers (CSW), and transgender people (TG) and their sexual partners accounted for almost all new infections (approximately 97%) in the MENA region in 2019 (UNAIDS, 2020). Drivers of HIV and hepatitis C virus among key populations include injecting drug use, partially intersecting with multiple and concurrent sexual partnerships, gender inequalities and violence, displacement, stigma, and discrimination. There is a strong possibility for hidden HIV and hepatitis C virus epidemics in the MENA region among key populations, particularly PWID, MSM, and CSW, and in prisons, with convergence into the general population via sexual transmission.

Prevention and treatment programmes in many MENA countries are not reaching sufficient numbers of key populations at high risk of HIV infection. Repressive and punitive approaches to drugs, criminalisation, and aggressive policing continue to discourage healthcare seeking, reinforce marginalization and stigmatization and perpetuate unsafe use of drugs in the MENA region (Al-Shazly & Tinasti, 2016; MENAHRA, 2020a; Sander et al., 2019). This has also created mass incarceration rates of people who use drugs (PWUD), with those who are incarcerated continuing to be vulnerable to drug use, drug injecting/introduction to injecting recorded in many prisons, and remaining underserved by harm reduction, prevention, and treatment services for infectious diseases (Dolan et al., 2015; Heijnen et al., 2016; Moazen et al., 2018; Sander et al., 2019; Van Hout & Aaraj, 2020). There is disproportionate impact on vulnerable and marginalized groups including women, racial and ethnic minorities, indigenous people, and foreign nationals (Sander et al., 2019). These unique drivers and vulnerabilities are now likely to be exacerbated by the COVID-19 pandemic.

In the MENA, it is often non-governmental organizations (NGOs) that are at the forefront of advocacy initiatives and service provision in the fields of HIV, harm reduction, and key populations. PWUD/PWID and other key populations are known to be more comfortable accessing services through NGO structures, as they remain to be criminalized populations in most countries of the region. The 2020 MENA Harm Reduction Association (MENAHRA) situation assessment reports that the MENA region has witnessed a slow but steady increase in the harm reduction response among countries of the region in general, with some positive changes in harm reduction policy visibility, provision, and coverage since 2016. Countries with the best examples of HIV and harm reduction responses in the region (such as Iran, Lebanon, Morocco, Pakistan, Tunisia) have an active number of NGOs who are primarily providing services to key populations and are involved in scaling up the response in coordination with governmental and other national stakeholders. In six countries out of the 20 (Iraq, Kuwait, Libya, Oman, Qatar, United Arab Emirates) including within the MENAHRA remit, there is either a non-existent or a very limited role that NGOs play in the HIV and harm reduction response. Coincidentally, these countries do not provide comprehensive harm reduction programs that are tailored for key populations.

It is likely that such gains in harm reduction advocacy, engagement, and support of key populations are threatened by the impact of COVID-19. Commentaries have recently underscored that whilst MENA states have responded to this public health crisis swiftly by closing borders, restricting population movement, and instigating curfews, human rights impacts were generally ignored under the guise of continued enforcement of repressive laws and new legislation (Thiry, 2020). Some recent editorials from the MENA region (Iran, Pakistan) have documented the situation regarding harmful drug use, viral transmission, and harm reduction responses in the MENA region and have called for increased policy maker vigilance in supporting continued responses (Alavi et al., 2020; Aslam, 2020; Deilamizade & Moghanibashi-Mansourieh, 2020). Iran for example has illustrated how efforts to mitigate the COVID-19 crisis among PWUD, included cessation of compulsory and residential addiction treatment admissions for a time, prisoners release schemes, and longer take-home doses provided to those on opioid agonist therapy (OAT). These mitigation measures however increased homeless populations and service users of community harm reduction programmes (Deilamizade & Moghanibashi-Mansourieh, 2020). Challenges in preventing COVID-19 transmission were subsequently identified and included emergence of new drug hot spots and a disrupted drug distribution network, difficulties in applying isolation measures and care of PWUD with COVID-19, insufficient capacity of shelters and drop-in centres, restricted peer support staff capacity and levels of volunteerism, shortages of medical supplies, and heightened stigma of PWID particularly those who are homeless labelled as ‘coronavirus moving bombs’ (Deilamizade & Moghanibashi-Mansourieh, 2020).

With exception of Iran, very little is known about MENA countries relating to their COVID-19 response and the situation of PWUD during this public health emergency. In late 2020, MENAHRA commissioned a rapid research study to assess the situation of PWUD/PWID in the MENA region and assess the impacts on harm reduction services during the height of the COVID-19 pandemic, in order to encourage the response planning, review, and adaption of frontline and specialist drug interventions.

## Methodology

MENAHRA is the first network on injecting drugs harm reduction in the MENA region covering 20 countries (Afghanistan, Algeria, Bahrain, Egypt, Iran, Iraq, Jordan, Kuwait, Lebanon, Libya, Morocco, Oman, Pakistan, Palestine, Qatar, Saudi Arabia, Syria, Tunisia, UAE, Yemen), with its secretariat in Beirut. MENAHRA also has built and supported three knowledge hubs (KHs) based in Iran (KH-INCAS), Lebanon (KH-SIDC), and Morocco (KH-ArRazi). The choice of 20 countries was based on a prioritization exercise that targets some indicators related to injecting drug practices among PWID and the prevalence of HIV in this community at the countries’ levels, in addition to many other indicators. This was not related to any regional classification made by the World Bank, WHO, or UNAIDS, with the exercise conducted in collaboration with a WHO representative and a group of regional experts from the MENA region.

The research was conducted in November 2020 and aimed to explore and describe the impact of COVID-19 on PWUD behaviours, risk mitigation measures, and how harm reduction services in the MENA region have adapted and, in some cases, re-configured to respond to their needs. A mixed method study design was developed, with design of instruments informed by two prior in-depth investigations conducted by authors in mid-2020, the 2020 MENAHRA situation assessment on harm reduction programming in the MENA region (MENAHRA, 2020a), and the MENAHRA, 2020a, 2020bAdvocacy Briefs report (MENAHRA, 2020b).

Two primary data collection exercises were conducted, consisting firstly of a circulated survey sent to regional and all country level professional and policy level focal points in the region (e.g. World Health Organization Regional Office for the Eastern Mediterranean, United Nations Office on Drugs and Crime Regional Office for the Middle East and North Africa, UNAIDS, National AIDS Programmes, national and local NGOs), so as to receive where possible an updated perspective on how key populations and harm reduction services are responding to the COVID-19 crisis. This survey was circulated as part of the global 2020 Harm Reduction International and 2020 MENAHRA situation assessment on harm reduction (MENAHRA, 2020a) and asked three open-ended questions:

- How has the COVID-19 situation affected harm reduction services in your country in terms of coverage, access, and available resources?
- Were there any government measures introduced to help service delivery?
- Please provide an example on how an organization changed their harm reduction service delivery to respond to the COVID-19 situation?

Secondly, a virtual focus group (Zoom) was conducted with the MENA Network of People Who Use Drugs (MENANPUD) focal points. This virtual consultation connected the PWUD focal points regionally and explored the situation and impact of COVID-19, with specific focus on their navigation of public health guidance and lockdown measures both in the community and in prisons, drug consumption behaviour and related risks, drug distribution changes, risk to health, responses, and challenges faced, in order to best understand their experience and to generate and identify optimal mitigation measures and strategies to support them.

Ethical considerations were in line with the MENAHRA institutional guidelines and protocols for conducting research with vulnerable populations and stakeholders. All participants were provided with information and provided informed verbal consent prior to participation; the FGD (90 min) was audio recorded with participant permission and was transcribed, and the facilitator (one person) also adopted a critical listening approach and made detailed notes.

Both textual data sources were analysed manually using a thematic analysis (TA) framework approach (Braun et al., 2019). This involved several key steps: (1) reading and rereading the open-ended text/transcriptions, individually and in pairs to note early ideas; (2) coding in a systematic and logical manner using a data-driven approach and paying attention to interesting concepts and ideas within the data; (3) organization of codes into corresponding groups using an iterative process in developing themes and subthemes; (4) refining and reviewing of themes by the team as a collective in terms of internal homogeneity and external heterogeneity, examination of coherence of patterns across themes, and development of a thematic map; and (5) final clear definition and naming of themes, with data extracts representing and articulating the essence of the theme and overall analysis. Triangulation occurred between data sources and across methods and generate a series of illustrative themes, and derived recommendations and priority areas for targeted advocacy at country and regional levels.

## Results

There were 20 professional stakeholder responses (Algeria: *n* = 3; Bahrain: *n* = 1; Egypt: *n* = 2; Iran: n = 2; Jordan: n = 1; Lebanon: n = 3; Morocco: n = 2; Pakistan: n = 1; Palestine: n = 2; Syria: *n* = 1; Tunisia: n = 1; Yemen: n = 1) to the regional survey representing 12 MENA countries and two regional responses. The virtual focus group discussion included 11 MENANPUD focal points (Bahrain: *n* = 1; Egypt: *n* = 2; Jordan: n = 2; Lebanon: n = 1; Morocco: *n* = 3; Pakistan: n = 2). The participants of the MENANPUD focal point focus group were mainly males with one female representative from Pakistan, noting the difficulty in engagement of female PWUD due to underlying cultural and social taboos that subject them to increased stigma and discrimination when compared to male PWUD.

### The Impact of COVID-19 on PWUD Communities

Across all 20 countries, and regionally, COVID-19 was reported to incur a severe impact on drug use and related risk and also the coverage, access, and resources of harm reduction services. Drug distribution networks were reported to be affected by movement restrictions and drug availability, leading to some stakeholders describing increased risk taking due to drug related withdrawals.

The situation of COVID 19 surely impacted harm reduction services and the lives of people who use drugs, mainly during the confinement period. We can cite certain observations noted in the field, namely: The difficulty of accessing PWUD to provide them with the means of prevention given the daily change of grouping sites for consumption according to the places where the product is available. [Morocco stakeholder]

The lockdown measures, in particular the closing of country borders, affected the availability of some drugs, after which some countries saw shifts among PWUD to other drugs. In Morocco, for instance, the closing of borders impacted the availability of heroin, and painkillers were difficult to access. In contrast, in Pakistan, the increased use of painkillers among youth was highlighted along with their accessibility as no prescriptions are needed for their purchase. In Jordan, PWUD resorted to *Joker* a synthetic cannabinoid substance that is synthesized in the country when borders were closed. In these instances, risk was described as not only referring to blood-borne virus transmission but now also COVID-19. A stakeholder from Morocco said;

During its visits, the field team noted that a large part of the beneficiaries were found in a withdrawal state due to the unavailability of the consumption product. The scarcity of the product has led to the sharing of the doses purchased between several people, without sharing the syringes, but without respecting the preventive measures at Covid-19 despite the efforts made to raise awareness. [Morocco stakeholder]

The economic impact of COVID on PWUD also presented increased risky behaviour such as resorting to stealing, drug dealing, or dropping out of OAT or other therapeutic treatment due to inability to afford the costs of treatment.

A lot of people I know that are drug users are now becoming drug dealers to be able to get their drugs. During COVID, it’s more difficult and therefore they have to resort to dealing, stealing and other issues to secure drugs [Lebanon MENANPUD focal point]

COVID affected the economic situation for everyone, but for PWUD - where will they get the money to buy drugs? Most PWUD get paid on a daily basis and COVID also impacted this – there was less work and so they were paid less. Even begging was affected because mosques were closed. A lot of people resorted to stealing as well during this time to ensure that they have the money. [Morocco MENANPUD focal point]

Out of the beneficiaries that were unable to afford treatment – a lot of them actually relapsed and resorted to drug use again. Some were selling in exchange for the substance that they use to be able to afford it. [Lebanon stakeholder]

In Morocco, an increase in cases of violation of the rights of people who use drugs, stigma, and discrimination (e.g. requiring special authorizations from the authorities to travel to methadone dispensing centres), alongside increased basic human needs (food aid, rent subsidy, housing supports) due to unemployment of PWUD was observed. Many PWUD were also unable to benefit from COVID state support due to lack of official documents, highlighting inequality issues that have been further exacerbated during the pandemic.

We had a support fund for COVID in Morocco, but most PWUD did not get the chance to receive this support because they do not have the official papers (national ID, national health card) to qualify. [Morocco MENANPUD focal point]

Moreover, marginalized homeless PWUD were reported to be arrested due to violating curfew and lockdown measures by being in the street, further increasing their risk for COVID by being detained in crowded detention centres.

Drug users are more at risk now because they are in the street and looking for drugs and more at risk for arrest from police because they are going out to get their drugs and are present more on the street [Bahrain MENANPUD focal point]

During COVID a lot of drug users arrested – not because they drug users but because they were sleeping on the street because they are homeless and there was a lockdown. [Morocco MENANPUD focal point]

A stakeholder from Palestine described the impact of stigma of PWUD during COVID-19 and how this impacted on harm reduction service capacity, often reliant on volunteers;

There was a significant impact on the provision of services, especially during the quarantine period and lockdown, increased the number of drug users who died, especially the homeless, and the flight of many volunteers due to stigma and fear of infection, because users are among the people most vulnerable to the virus because of their way of life. Also, the services that are provided to them are limited to distributing needles and some clothes [Palestine stakeholder]

### Harm Reduction Service Coverage, Access, and Available Resources

In many countries, functioning and operations of harm reduction and the accessing of hospital care during curfews/lockdown were affected. Many observed that social distancing made it impossible for PWUD to seek and access services.

It is a disaster for the PWUDs; population heavily penalized by law and who is completely outside the state protection circuits. [Algeria stakeholder]

Harm reduction services have been affected - less services are provided. Also, as going out is not as easy as before [confinement measures], access to services has been affected. We aren’t allowed to go out due to COVID and it is difficult to contact with others. PWUD are accessing services only weekly. [Pakistan MENANPUD focal point]

In Yemen the situation was reported to be dire.

Since the beginning of the spread of Covid-19 in Yemen, in Aden in particular, no organization or government has bothered to reduce the harm, as many people die… women and men without being accepted by any hospital or clinic with chronic diseases and others. We are concerned for the most marginalized and vulnerable groups. [Yemen stakeholder]

COVID-19 testing services were also not as accessible to the PWUD/PWID population mainly due to the underlying stigma and discrimination issues that they are faced with. This has led the PWUD community to request COVID testing services in drug user friendly facilities as well as in mobile health units.

PWUD did not get the chance to get a COVID test because a lot of people go there to get tested and PWUD are stigmatized against and therefore they avoid going to health centers. [Morocco MENANPUD focal point]

COVID testing for PWUD specifically should be provided. [Pakistan MENANPUD focal point]

Others observed that restrictions also impacted on staff capacity to provide services.

Social distancing prevented people from seeking services. Due to confinement, centres and staff were limiting their working days and thus limiting the time for service delivery. [Jordan stakeholder]

Some treatment centres – 12 steps - closed [during COVID] and people were in the street. The governmental centres let people go or they were released to other centres. [Bahrain MENANPUD focal point]

The unavailability of certain drugs and inability of hospitals to meet the increased demand for treatment services for PWUD due to COVID led to legal consequences in Morocco, where PWUD were arrested during demonstrations against hospitals when they were seeking treatment due to decreased availability of heroin and painkillers.

Painkillers during COVID were difficult to find, causing PWUD to seek treatment in hospitals but with no help because hospitals were overwhelmed. This caused demonstrations which led to arrest. [Morocco MENANPUD focal point]

State level supports to continue harm reduction service delivery to key populations were generally observed to be insufficient during the COVID-19 pandemic, with initiatives backfilled by UN agencies (UNAIDS, UNODC). UNODC contributed to COVID-19 pandemic in the MENA region by disbursing developed English UNODC infographics and information on COVID-19 detailing prevention measures for people who use drugs and people living in closed settings in Arabic and French and through procurement of personal protective equipment for the prisons and prison health staff in Egypt, Morocco, and Tunisia and organized rounds of virtual training to the prison staff of the region.

In some countries such as Morocco, government initiatives supported the resourcing for medical masks and hydroalcoholic gels for members of the harm reduction field team. Many governments extended the visit intervals with service users to reduce exposure and at the same time avoid medication shortage. Most stakeholders described the subsequent negative effect on viral testing of PWUD, sexually transmitted infection testing, counselling and provision of prevention information and clean needles. Medicine provision for PLHIV was generally reported to continue routinely.

Special support from multilateral partners (UNAIDS and UNODC) for program continuity. Many initiatives not always coordinated for “all” but no specific programs for key populations including PWUDs. [Algeria stakeholder]

In Iran, government support was activated to include harm reduction settings in the COVID-19 response:

Government supported harm reduction centers for providing free of charge masks, gloves, disinfectants …Yes, through donating supplies and direct funding to purchase the necessary supplies. [Iran stakeholder]

A number of suggestions and recommendations were made by PWUD representatives in terms of service needs and improvements. An emphasis on the provision of COVID testing for PWUD was made, due to concerns that PWUD often avoid health clinics and hospitals because of stigma and discrimination.

PWUD do not go to testing centers and wait in line and are scared of discrimination. COVID testing needs to be available in drug user friendly centers or in a mobile unit. [Morocco MENANPUD focal point]

The need for increased accessibility to harm reduction services, whether already available in the country or not, was also noted, illustrating the importance of dedicating efforts to scaling up and introducing harm reduction services.

Making methadone available to everyone is important because if this was available there would be no black market. [Morocco MENANPUD focal point]

NSPs should distribute more frequently. Naloxone should be available without any discrimination as well as overdose prevention. OAT should be legalized in Pakistan. [Pakistan MENANPUD focal point]

Moreover, PWUD indicated that there is a need for interventions addressing the economic situation of PWUD, including provision of homeless centres, food aid, employment services support, and legal support to address issues related to drug use on the criminal record that serve as obstacles for employment.

They also need to provide homeless centers for PWUD because most of them are homeless, and this increased their arrests during lockdown and put them at risk. [Morocco MENANPUD focal point]

Having a job gives you the confidence and is important. In the law as a drug user, if I have a legal problem, the center I go to lacks legal services and we need this to help secure jobs. [Lebanon MENANPUD focal point]

### Community Outreach, Mobile, and Technology-Assisted Means

Most national stakeholders described revision of working practices and development of new protocols for supporting harm reduction service users during COVID-19. Some countries reported use of telemedicine to continue contact with their service users (counselling, prevention awareness raising) and flexible operations regarding OAT dispensing and service user supports. Some described new protocols for service operations, and targeted prevention materials for PWUD.

We (civil society) with the support of UNAIDS, UNODC and the Ministry of Health have adopted an approach adapted to the context which takes into account the difficulties caused by COVID-19.- This included re-organization of field teams, change of working hours, training of workers on COVID-19 prevention instructions, protection and communication equipment, adaptation of the monitoring and evaluation (M&E) system, interruption of screening except for emergencies, and implementation of a new guidance and support system. [Algeria stakeholder]

Due to COVID-19 pandemic, services in Morocco and Afghanistan were readapted to allow up to 2-week take-home doses of methadone, home delivery and larger supply of syringes for PWID drugs- to minimize crowding at health centers -as part of social distancing, overcome the difficulties of commuting during lockdown and ensure uninterrupted service delivery. [Regional level stakeholder]

Protocols were drafted in response to COVID-19 so that maintenance treatment medications were conducted through flexible take-home forms and replace in-persons visits with visits via telephone or video call. [Iran stakeholder]

The government, as well as, civil society are encouraging virtual delivery of services using different means including webinars and modern media. [Egypt stakeholder]

We [NGO] activated the hotline for services so people could call at any time if they have any question – this included online counselling and therapy as well as medical consultations and advice. [Lebanon stakeholder]

Morocco in particular responded via several measures: virtual training of staff, purchase of prevention equipment (surgical masks, disposable gloves, and hydroalcoholic gel), field actions to raise awareness among beneficiaries and the distribution of prevention tools while insisting on compliance with barrier measures; community mobilization; extension of the opening hours of the low-threshold centre to provide beneficiaries with social benefits on an individual basis and absolute compliance with preventive measures, food aid; and meetings on virtual platforms. All reported adjusting the schedule of field work in line with curfew schedules. The shift toward community outreach support of PWUD in hot spots by mobile harm reduction services was described by all stakeholders. OAT in many countries continued via community distribution and via use of mobile health units. Harm reduction teams not only focused on known drug hot spots but also delivered methadone in the community and in prisons and maintained contact with PWUD via *WhatsApp*. They also provided disinfectants and masks, alongside needle and syringe exchange.

Social distancing made the admission of the centres decrease but mobile van and outreach services expanded. [Iran stakeholder]

We have set up a mask manufacturing unit entirely managed by female PLHIV. The masks are intended for key populations including the PWUDs. [Algeria stakeholder]

In some countries, anti-retroviral treatment (ART) supports specifically for PWUD in clinic form were halted with ART provision continued via home delivery. In Bahrain, an online system to arrange home delivery of medicines was created for PLHIV. In Palestine, a stakeholder described:

Cooperation with the national security forces to deliver treatment to the patients at their home. The role of the government was limited to giving us a freedom of transportation permit. [Palestine stakeholder]

There were some positive shifts in OAT medicine regulation during the COVID-19 pandemic. In some countries such as in Pakistan, endorsement of OAT in Pakistan by the Drug Regulatory Authority and Anti-narcotic Forces and steps toward registering methadone and buprenorphine reached advanced stages. Similarly, in Egypt, as part of elimination of hepatitis C, the government requested WHO support for the introduction of OAT. Other countries described development of new working groups and committees to support the COVID-19 response for PWUD. In Lebanon, the Ministry of Health (through an official circular) has allowed the dispensing of buprenorphine for 2-week intervals for people on OAT as one of the measures for COVID-19. This was not possible previously (there was only dispensing at 1-week intervals). Further in Lebanon, there were efforts to support those in prison and efforts to activate release of PWUD arrested for drug use charges to avoid further overcrowding in pre-trial detention centres and police stations. When NGOs that followed-up on OAT within prison were restricted from accessing the prison due to COVID measures, the prison medical doctor reached out to them to maintain urine testing and other required follow-up services to ensure that prisoners continued to receive their medication.

## Discussion

We present here the first collective regional effort to describe the situation of PWUD in the MENA as a regional experience and how COVID-19 impacted on the situation of PWUD, their ability to mitigate against COVID-19, and the delivery and configuration of harm reduction services. Ultimately findings illustrate the challenges encountered by PWUD and how harm reduction services in some countries rapidly responded to their needs. Our regional investigation whilst small scale was conducted during a time of public health (and humanitarian in some countries) crisis and compliments efforts documented at the global level. There is growing concern highlighted in the most recent World Drug Report (UNODC, 2020) of an increase in harmful patterns of drug use, whether through a switch to injecting or through more frequent injecting among users, as a result of the COVID-19 pandemic. An increased risk of contracting COVID-19, as well as other diseases such as HIV and HCV, coincides with expected increases in injecting drug use and sharing of injecting equipment due to drug shortages or safe injecting material shortages (UNODC, 2020). It has been observed that substance use treatment and harm reduction services are severely impacted during the pandemic, with recommendations centring on the requirement for policy makers to have business continuity plans in place and ensure continued use of evidence-based intervention for PWUD, with sufficient medicine supplies, with harm reduction integrated within other treatment modalities, and with specific focus on particularly vulnerable groups such as refugees and immigrants (Radfar et al., 2020). Global guidance documents on the comprehensive health system response have been generated, with a specific focus on the rights of vulnerable groups, and centre on protection of staff, screening of PWUD with COVID respectful of human rights, and the use of innovative technology-based solutions (virtual, mobile-based platforms) to support interventions (Farhoudian et al., 2020).

The study underscores the important and vital role of NGOs in the COVID-19 response to PWUDs. Civil society engagement and the work of NGOs in the MENA region continue to represent the key drivers of the HIV and harm reduction and now COVID-19 response (MENAHRA, 2020a). Some are hampered by ongoing conflict and humanitarian crisis. The findings are illustrative of the rapid ability of some MENA countries with available harm reduction services (despite lack of state support) to swiftly adapt their services to help prevent COVID-19 transmission in PWUD networks but also ensure continuity of harm reduction programming and treatment for PLHIV. Requirements to ensure social distancing of staff, volunteers, and service users have created increased demands on harm reduction service programming. Innovative approaches have included shifting counselling services to online platforms for psychosocial counselling, extending validity of OAT prescriptions, distribution of hygiene and personal protective equipment to PWUD and healthcare workers, distribution of harm reduction and safe injecting materials in larger quantities to decrease frequency of visits, as well as meeting other emergent needs and connecting with PWUD via mobile outreach. Shortages of opioids due to COVID-19 restrictions, as well as a deteriorating financial situation as a result of these restrictions, were among the concerns highlighted that may impact PWUD. During COVID-19, PWUD in the MENA face the same risks as those of the general population and therefore need to be sensitized and aware of the appropriate advice to reduce their risk of infection. They can be exposed to additional risks, which are more likely increased by the high level of physical and psychological comorbidity found among some PWUD, the fact that drug problems are often more common in marginalized communities, and the stigmatization that these key populations often experience. Several factors that elevate key population risk of HIV acquisition may also place them at higher risk of acquiring COVID-19, such as high mobility and close physical contact with others through social and sexual practices.

Triangulation of findings highlights the need for reinforced advocacy efforts within the MENA region, and at country levels, and expands them to include tailored services for PWUD within the broader COVID-19 pandemic response. Information collected can further inform national and local level service reviews and updates within the context of country-specific guidelines and rules for responding to the COVID-19 outbreak and generate recommendations regarding optimal mitigation strategies for PWUD and drug service providers during COVID-19 in the MENA region and elsewhere. In countries where the harm reduction response is non-existent, the continuation and reinforcement of advocacy efforts to support policy reform, recognition of PWUD as key populations, and the initiation of PWUD services are recommended. In countries where harm reduction services do exist, the interruption to harm reduction services, capacity issues of staff, difficulty in accessing services due to lockdowns and other underlying issues such as stigma and discrimination, as well as risk of interruption of OAT medication stocks are all concerns highlighted. Therefore, there is an imperative for further advocacy to ensure quality of care for PWUD. Inclusion in domestic emergency planning is warranted to ensure integration of harm reduction services as an essential health service spanning the community and prison responses. This is advised to include the development of contingency plans that address continuity of harm reduction services (including ART and OAT supplies) during health emergencies and disease outbreaks, with tailored guidelines and protocols approved by the relevant governmental entities to allow for exceptions for provision of such services during any government restrictions. NSP and naloxone distribution through drop in centres and other health centres, mobile health units, and street outreach to known drug hot spots are advised to continue and with support through telemedicine. The government COVID-19 response must include COVID-19 testing and support services for PWUD and fully utilize and resource existing NGOs and other centres that are PWUD-friendly to ensure access and the coverage of the PWUD population. Further, it must include provision of food and economic support aid for PWUD during disease outbreaks and channelled through existing NGOs. Policy reforms during such health emergencies are also warranted to include PWUD in prison release schemes, and the referral of PWUD to support structures if homeless, violating restrictions such as lockdown or curfew measures.

## Conclusion

The study highlights how COVID-19 aggravated existing marginalization and stigmatization of PWUD and other key populations in the MENA region, with government level resourcing severely impacted by COVID-19. It further illustrates the commitment by harm reduction NGOs in diversifying their response, particularly through mobile outreach to drug hot spots, and the reliance of technology to support awareness raising, telemedicine, and medicine supplies. It is encouraging to observe that in some MENA countries, positive shifts are observed in harm reduction policy by government and the continued commitment to support PWUD communities by existing harm reduction NGOs. Continued advocacy for and implementation of harm reduction responses at the domestic and regional levels should be underpinned by inclusion in state health emergency planning and disease control efforts, awareness raising around innovation and telemedicine to support health and NGO support systems and medicine supply chains, resourcing of NGOs, and provision of economic support for PWUD, disease surveillance, and research.
